# The combined transduction of copper, zinc-superoxide dismutase and catalase mediated by cell-penetrating peptide, PEP-1, to protect myocardium from ischemia-reperfusion injury

**DOI:** 10.1186/1479-5876-9-73

**Published:** 2011-05-21

**Authors:** Guang-Qing Huang, Jia-Ning Wang, Jun-Ming Tang, Lei Zhang, Fei Zheng, Jian-Ye Yang, Ling-Yun Guo, Xia Kong, Yong-Zhang Huang, Yong Liu, Shi-You Chen

**Affiliations:** 1 Institute of Clinical Medicine and Department of Cardiology, Renmin Hospital, Hubei University of Medicine, Shiyan, Hubei 442000, China; 2 Department of Critical Care Medicine, Renmin Hospital, Hubei University of Medicine, Shiyan, Hubei 442000, China; 3 Department of Physiology and Key Lab of human Embryonic Stem Cell of Hubei Province, Hubei University of Medicine, Hubei 442000, China; 4 Department of Physiology & Pharmacology, The University of Georgia, Athens, GA 30602, USA

## Abstract

**Background:**

Our previous studies indicate that either PEP-1-superoxide dismutase 1 (SOD1) or PEP-1-catalase (CAT) fusion proteins protects myocardium from ischemia-reperfusion-induced injury in rats. The aim of this study is to explore whether combined use of PEP-1-SOD1 and PEP-1-CAT enhances their protective effects.

**Methods:**

SOD1, PEP-1-SOD1, CAT or PEP-1-CAT fusion proteins were prepared and purified by genetic engineering. *In vitro *and *in vivo *effects of these proteins on cell apoptosis and the protection of myocardium after ischemia-reperfusion injury were measured. Embryo cardiac myocyte H9c2 cells were used for the *in vitro *studies. *In vitro *cellular injury was determined by the expression of lactate dehydrogenase (LDH). Cell apoptosis was quantitatively assessed with Annexin V and PI double staining by Flow cytometry. *In vivo*, rat left anterior descending coronary artery (LAD) was ligated for one hour followed by two hours of reperfusion. Hemodynamics was then measured. Myocardial infarct size was evaluated by TTC staining. Serum levels of myocardial markers, creatine kinase-MB (CK-MB) and cTnT were quantified by ELISA. Bcl-2 and Bax expression in left ventricle myocardium were analyzed by western blot.

**Results:**

*In vitro*, PEP-1-SOD1 or PEP-1-CAT inhibited LDH release and apoptosis rate of H9c2 cells. Combined transduction of PEP-1-SOD1 and PEP-1-CAT, however, further reduced the LDH level and apoptosis rate. *In vivo*, combined usage of PEP-1-SOD1 and PEP-1-CAT produced a greater effect than individual proteins on the reduction of CK-MB, cTnT, apoptosis rate, lipoxidation end product malondialdehyde, and the infarct size of myocardium. Functionally, the combination of these two proteins further increased left ventricle systolic pressure, but decreased left ventricle end-diastolic pressure.

**Conclusion:**

This study provided a basis for the treatment or prevention of myocardial ischemia-reperfusion injury with the combined usage of PEP-1-SOD1 and PEP-1-CAT fusion proteins.

## Introduction

Ischemic heart disease, especially acute myocardial infarction (AMI), a primary myocardial disease characterized by the loss of cardiomyocytes and the increase of fibroblasts, is an important cause of heart failure. Early reperfusion is an absolute prerequisite for the survival of ischemic myocardium. However, reperfusion has been referred as the "double-edged sword" because reperfusion itself may lead to accelerated and additional myocardial injury beyond that generated by ischemia, which results in a spectrum of reperfusion-associated pathologies, collectively called reperfusion injury [1].

Several mechanisms have been proposed to cause reperfusion injury including formation of oxygen free radicals (OFR), calcium overload, neutrophils-mediated myocardial and endothelial injury, progressive decline in microvascular flow to the reperfused myocardium, or depletion of the high-energy phosphate store [2]. Among these factors, overproduction of OFR during the first few minutes of reperfusion is considered as a key event. About 25% of cell death in cardiomyocytes after reperfusion of acute myocardial infarction is caused by reperfusion injury [3]. OFR includes superoxide anion (O_2_^-^), hydroxyl radical (OH^-^), hydrogen peroxide (H_2_O_2_), etc. Excessive OFR causes cell DNA breakage, degeneration, and lipid peroxidation, ultimately leading to cell death. The key antioxidant enzymes, including superoxide dismutase (SOD), catalase (CAT) and glutathione peroxidase (GPx), provide a defense system against oxidative stress by removing the OFR, thus protecting cells from oxidative damage [4,5]. However, the endogenous antioxidant activity is severely damaged after ischemia-reperfusion which makes the myocardium extremely vulnerable to OFR [6]. Moreover, exogenous SOD1 and CAT can not be delivered into living cells because of the poor permeability and selectivity of the cell membrane, which has limited its usage in protecting cells/tissues from oxidative stress damage.

There is a growing effort to circumvent these problems by designing strategies to deliver full-length proteins into a large number of cells. Morris Group [7] designed and synthesized a new type of cell penetrating peptide PEP-1, which consists of three domains: a hydrophobic tryptophan rich motif (KETWWETWWTEW), a spacer (SQP), and a hydrophilic lysine-rich domain (KKKRKV). Pep-1 is cationic and adopts amphipathic a-helical structure on the membrane. These characteristics are similar to those of cationic antimicrobial peptides involved in host innate immunity, suggesting that PEP-1 can kill microbes [8-10]. In addition, Park's study [11] shows that PEP-1 has antichlamydial activity. More importantly, many studies have demonstrated the successful delivery of full-length PEP-1 fusion proteins into cultured cells and the nervous system by protein transduction technology including EGFP, β-Gal, full-length specific antibodies, human copper chaperone for Cu, Zn-SOD, CAT and SOD [7,12,13]. Our previous studies indicate that PEP-1-SOD1 or PEP-1-CAT fusion proteins can be transduced into myocardial tissues to protect myocardium from ischemia-reperfusion-induced injury in rats [12,14]. Cu, Zn-superoxide dismutase (Cu, Zn-SOD, also called SOD1) only catalyzes the dismutation of O_2_^- ^into H_2_O_2 _and O_2_. Elimination of H_2_O_2 _requires the endogenous CAT or GPx activity, which removes H_2_O_2 _by breaking it down into H_2_O and O_2_, thus preventing the generation of OH^-^. Therefore, we hypothesize that combination of PEP-1-SOD1 and PEP-1-CAT fusion proteins is more useful in preventing myocardium from ischemia-reperfusion injury.

## Materials and methods

The present study conformed to the Guide for the Care and Use of Laboratory Animals published by the US National Institutes of Health (NIH Publication number 85-23, revised 1985). The animal use protocol was approved by the Institutional Animal Care and Use Committee of Hubei University of Medicine.

### Expression, purification and transduction of PEP-1-SOD1 and PEP-1-CAT

Four prokaryotic expression plasmids with His-tag, pET15b-SOD1-His, pET15b-PEP-1-SOD1-His, pET15b-CAT-His, and pET15b-PEP-1-CAT-His were constructed by the TA-cloning method. The recombinant plasmids were transformed into *E.coli *BL21 (DE3) (Novagen, USA). The transformed bacteria were grown in 100 ml LB medium at 37°C to an OD600 value of 0.5-1.0 and induced with 0.5 mM isopropyl-β-D-thiogalactoside (IPTG) (Promega, USA) at 25°C for 12 h. Bacteria were lysed by sonication at 4°C in a binding buffer (5 mM imidazole, 500 mM NaCl, 20 mM Tris-HCl, pH 7.9). To purify the recombinant fusion proteins, cell lysates were loaded onto a Ni^2+^-nitrilotriacetic acid sepharose affinity column (Qiagen, USA) under native conditions. After the column was washed with 10 volumes of the binding buffer and 6 volumes of wash buffer (60 mM imidazole, 500 mM NaCl, 20 mM Tris-HCl, pH 7.9), the fusion proteins were eluted using an eluting buffer (1 M imidazole, 500 mM NaCl, 20 mM Tris-HCl, pH 7.9). The fusion-protein-containing fractions were combined, and the salts were removed using a PD-10 column. Protein concentrations were measured by the Bradford method [15].

### Cell culture

H9c2 cells, derived from embryonic heart tissue (American Type Culture Collection, Manassas, VA), were cultured in Dulbecco's modified Eagle's medium (DMEM, Invitrogen) with 5 g/L glucose supplemented with 15% (v/v) fetal bovine serum (FBS, Hangzhou Sijiqing Biological Engineering Materials Co. Ltd., China). Cells were routinely grown to subconfluency (> 90% by visual estimate) in 75 cm^2 ^flasks at 37°C in a humidified atmosphere of 5% CO_2 _prior to passage and seeding for experiments.

### Transduction of PEP-1-SOD1 and PEP-1-CAT fusion protein into H9c2 cells

H9c2 cells were grown to confluence on 25 cm^2 ^flasks and pretreated with PEP-1-SOD1-His or PEP-1-CAT-His at different doses (0.5~2.0 μM) for 15 min~72 h. The cells were then washed with phosphate-buffered saline (PBS) and treated with trypsin-EDTA followed by lysate preparation for western blot or enzyme activity assay. The SOD and CAT activity were measured using SOD and CAT kits by following the manufacturer's protocols (JianCheng Bioengineering Institute, China).

### Immunocytochemistry

To directly visualize the transduction of PEP-1-SOD1 and PEP-1-CAT fusion protein into H9c2 cells, cells were treated with 2 μM of control SOD1, purified PEP-1-SOD1, CAT, or PEP-1-CAT. After 1 or 6 h of incubation at 37°C, the cells were washed twice with 1 × PBS and fixed with 4% paraformaldehyde for 15 min at room temperature. Immunocytochemistry was performed by incubation with specific primary antibodies: rabbit anti-polyhistidine (diluted 1:200) (Santa Cruz Biotechnology, USA) or mouse anti-Troponin T (diluted 1:200) (Santa Cruz Biotechnology, USA) at 4°C overnight. Cells were then incubated with TRITC-conjugated rat anti-rabbit Ig G (diluted 1:250) or FITC-conjugated goat anti-mouse Ig G (diluted 1:250) at 25°C for 2 h. Nuclei were stained with DAPI (Sigma, USA). The immunoreactions were observed under a fluorescent microscope (Nikon, Japan).

### Hypoxia-reoxygenation treatment of H9c2 Cells

For the protective effect of combined pretreatment of PEP-1-SOD1 and PEP-1-CAT on H9c2 cells, cells were pretreated with or without PEP-1-SOD1 (2 μM) for 1 h or PEP-1-CAT (2 μM) for 6 h. Then, medium was changed to DMEM containing 1 g/L glucose and 1% FBS. Cells were cultured in a humidified hypoxia chamber (Stem Cell Technology, USA) and flushed with 95% N_2 _+ 5% CO_2 _to achieve 0.1% oxygen environment. The sealed chamber was placed into a 37°C incubator for 21 h. After hypoxia incubation, the cells were reoxygenized with fresh medium and incubation in 95% air + 5% CO_2 _for 6 h [16]. Control cells were kept in normoxic conditions for the corresponding times. The supernatants and cells were collected respectively after treatment.

### Annexin V and propidium iodide (PI) binding assay

To measure H9c2 cell apoptosis after hypoxia-reoxygenation treatment, we labeled the cells with Annexin V and PI fluorescein (Bender MedSystems, Austria). Cells were washed with 1×PBS, and suspended in 200 μl 1×binding buffer (10 mM HEPES pH 7.4, 140 mM NaCl, 2.5 mM CaCl_2_)/1×10^6^/L cells. Cells were then incubated with Annexin V (1:20) for 3 min followed by PI for 15 min. The apoptosis rate was evaluated by Flow cytometry.

### Transduction of PEP-1-SOD1 and/or PEP-1-CAT in rat myocardium and ischemia-reperfusion injury

To observe whether transduced PEP-1-SOD1 and PEP-1-CAT protect myocardial ischemia-reperfusion injury *in vivo*, we established the model of myocardial ischemia-reperfusion injury in rats. 240-280 *g *male Sprague-Dawley rats were obtained from the Experiment Animal Center at Hubei University of Medicine and housed at an appropriate temperature (25°C) and relative humidity (55%) with a fixed 12 h light/dark cycle and free access to food and water. The animals were randomly divided into five groups as follows: sham-operated group, ischemia-reperfusion injury group (I/R), PEP-1-SOD1 pretreatment (2 mg/Kg), PEP-1-CAT pretreatment (2 mg/Kg), and PEP-1-SOD1 (2 mg/Kg) + PEP-1-CAT (2 mg/Kg) pretreatment (n = 20 for each group). The animals were anesthetized with 10% chloral hydras (250 mg/kg, i.p.) and ventilated during the LAD coronary artery ligation. Surgery was performed under sterile conditions. One hour after pretreatment with PEP-1-SOD1 and/or PEP-1-CAT (i.p.), the left anterior descending coronary artery (LAD) was ligated for one hour followed by two hours of reperfusion as described previously [12,14].

### Measurement of creatine kinase (CK), CK-MB activity, cardiac troponin T (cTnT), and malondialdehyde (MDA) levels

Rat serum was obtained after centrifugation of blood samples at 3,500 rpm for 15 min. CK activities were measured by spectrophotometry at 340 nm [12]. Malondialdehyde (MDA), an end product of peroxidation of cell membrane lipids caused by OFR, is considered as a reliable marker of cardiomyocyte oxidative damage. MDA level was determined by measuring chromogen generation from the reaction of MDA with 2-thiobarbituric acid. The CK and MDA biochemical analyses were performed using commercial kits (JianCheng Bioengineering Institute, China). CK-MB activity and cTnT levels were quantified by ELISA (Rapidbio, USA).

### Western blot

Rat hearts were transected along the LAD ligature to separate ischemic tissue and remote myocardium. Heart tissues were lysed in lyses buffer. Western blot analysis was performed using procedures established in our laboratory. The following antibodies were used: rabbit anti-Bax (Santa Cruz Biotechnology), mouse anti-Bcl-2 (Santa Cruz Biotechnology), and peroxidase-conjugated secondary antibody (Sigma). The bands were visualized using the enhanced chemiluminescence (Sigma).

### Measurement of hemodynamics

Hemodynamic measurement was performed as described previously [12,14]. Briefly, after 2 hours of reperfusion, left carotid artery and femoral artery were exposed. Two catheters filled with heparinized (10 U/ml) saline solution were connected to a Statham pressure transducer (Gould, Saddle Brook, USA). The carotid arterial catheter was advanced into the left ventricle to record ventricular pressure for 3~5 min. The femoral artery catheter was inserted into an isolated femoral artery to monitor hemodynamics. Hemodynamic parameters were monitored simultaneously and recorded on a thermal pen-writing recorder (RJG-4122, Nihon Kohden, Japan) and on an FM magnetic tape recorder (RM-7000, Sony, Japan).

### Evaluation of infarct Size

Six or seven hearts in each group were used for this experiment. After 2 hours of reperfusion, the hearts were removed and treated with K-H buffer at room temperature for 3 minutes, and then frozen at -20°C for 1 h followed by transverse sectioning into 4 parts (thickness, 2-5 mm). Sections were incubated in 1% 2, 3, 5-triphenyltetrazolium chloride (TTC) at 37°C for 15 minutes. TTC did not stain the infarcted myocardium, thus showing white in color while non-ischemic myocardium was stained by TTC and showed brick-red in color. In the ischemia-reperfusion hearts, the left ventricle was at risk of infarction, the total and infarcted areas of left ventricle were measured using planimeter in a double-blinded manner. The volumes of the infarcted zone were calculated by multiplying the planimetered areas by slice thickness. Infarcted volume was expressed as the percentage of left ventricular volume for each heart.

### Statistical analysis

All data are expressed as means ± SD. Differences between groups were determined with unpaired Student t-test and one-way analysis of variance followed by a Newman-Keuls post hoc test. Probability values of *P *< 0.05 were considered to be significant.

## Result

### Expression and purification of PEP-1-SOD1 and PEP-1-CAT fusion protein

**Figure 1:**
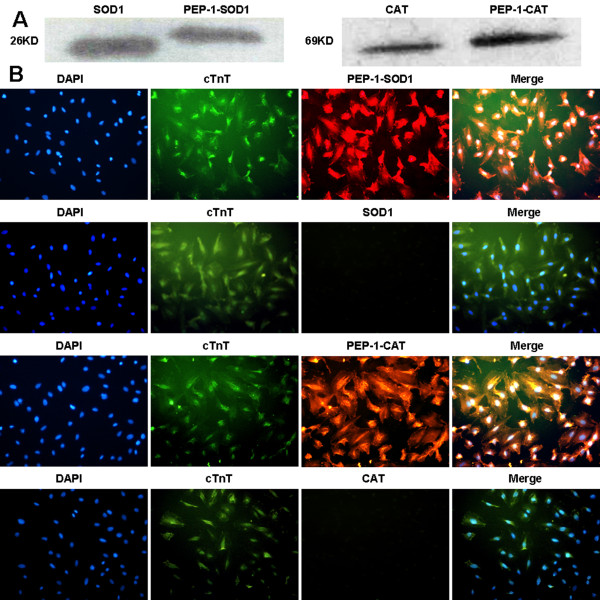
**Transduction of purified PEP-1-SOD1 or PEP-1-CAT into H9c2 cells**. A: purification of PEP-1-SOD1-His and PEP-1-CAT-His fusion proteins. Purified fusion proteins were analyzed by western blot with rabbit anti-polyhistidine antibody. B: H9c2 cells were treated with 2 μM purified His-tagged PEP-1-SOD1, wild type SOD1, PEP-1-CAT, or wild type CAT proteins for 6 h. Cells were incubated with rabbit-anti-polyhistidine and mouse-anti Troponin T (cardiomyocyte marker) antibodies (cTnT), and then visualized with fluorescent microscopy. Red fluorescent signals represent TRITC-labeled His-tag of SOD1 or CAT, Green fluorescent signals represent FITC-labeled Troponin T; Blue fluorescent signals represent DAPI-labeled nuclei.

pET15b-SOD1-His, pET15b-PEP-1-SOD1-His, pET15b-CAT-His and pET15b-PEP-1-CAT-His were successfully expressed and purified as shown in Figure 1A. The results indicated that the purified proteins had the correct molecular mass: i.e., SOD1, 22 KDa; PEP-1-SOD1, 26 KDa, CAT and PEP-1-CAT: 69 KDa. In addition, their enzyme activities were 356.98 U/mg, 355.54 U/mg, 3.18×10^3 ^U/g, 3.22×10^3 ^U/g, respectively. These data suggest that fusion proteins PEP-1-SOD1-His or PEP-1-CAT-His had the similar enzymatic activities as the wild type SOD1 or CAT.

### Transduction of PEP-1-SOD1 or PEP-1-CAT into H9c2 cell

The subcellular transduction of PEP-1-SOD1 or PEP-1-CAT fusion protein into H9c2 cells was confirmed by direct fluorescence analysis. As shown in Figure 1B, almost all cultured cells were transduced with PEP-1-SOD1 or PEP-1-CAT fusion proteins. However, the red fluorescent signals were not detected in cells treated with control SOD1 or CAT.

**Figure 2:**
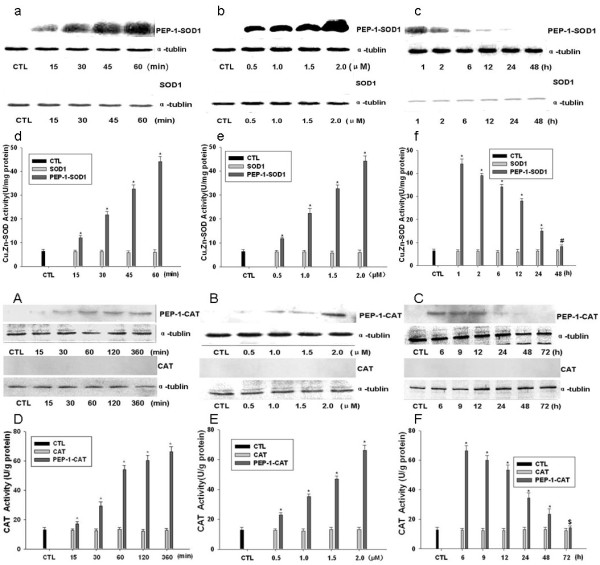
**Transduction and enzyme activities of PEP-1-SOD1 and PEP-1-CAT fusion proteins in H9c2 cells**. (a-c): Time and dose-dependent transduction of PEP-1-SOD1. Control or 2 μM SOD1 was added into the culture medium for 15~60 min (a-Short time course); 0.5~2 μM PEP-1-SOD1 or control SOD1 was added to the culture medium for 1 h (b-dose dependent); or cells pretreated with 2 μM PEP-1-SOD1 were incubated for different times (1~48 h) (c-longer time course). Western blots were performed using anti-His antibody. (A~C): Time and dose-dependent transduction of PEP-1-CAT. 2 μM PEP-1-CAT or control CAT was added into the culture medium for 15~360 min (A-shorter time course); 0.5~2 μM PEP-1-CAT or control CAT was added to the culture medium for 6 h (B-dose-dependent); or cells pretreated with 2 μM PEP-1-CAT were incubated for different times (6~72 h) (C-longer time course). Western blots were performed using anti-His antibody. (d~f): Enzymatic activity of PEP-1-SOD1. (D~F): Enzyme activity of PEP-1-CAT. Results are mean ± SD, n = 5, *P < 0.01, ^#,$ ^*P *< 0.05 vs control (CTL) group in each individual set of experiments.

To further investigate the transduction efficiency of PEP-1-SOD1 and PEP-1-CAT fusion proteins, we incubated H9c2 with 2 μM of PEP-1-SOD1 or PEP-1-CAT fusion proteins in cell culture medium at different time intervals, and analyzed the cellular fusion protein levels by western blotting. The intracellular fusion proteins were detected within 15 min and gradually increased until 60 min (PEP-1-SOD1) or 360 min (PEP-1-CAT) (Figure 2a and 2A). Moreover, the fusion proteins were transduced into H9c2 cells in a dose-dependent manner (Figure 2, b, c, B, C). The wild type SOD1 or CAT was not transduced into the cells (Figure 2)

It is essential that transduced PEP-1-SOD1 or PEP-1-CAT fusion proteins in cells retain their enzymatic activity. Therefore, we detected the SOD1 or catalase activities. As shown in Figure 2, the enzymatic activity of SOD1 or CAT in transduced cells increased in a dose- and time-dependent manner. Nearly seven (SOD1) or five fold (CAT) increase was observed in groups treated with PEP-1-SOD1 (Figure 2, d, e, f) or PEP-1-CAT (2 μM) (Figure 2, D. E, F), but not with the control SOD1 or CAT. These results demonstrate that the PEP-1-SOD1 or PEP-1-CAT fusion proteins were not only able to be transduced into H9c2 cells, but also was the transduced proteins able to retain their enzymatic activities for at least 48 h.

### PEP-1-SOD1 and PEP-1-CAT decreased LDH levels and inhibited H9c2 cell apoptosis in vitro

**Figure 3:**
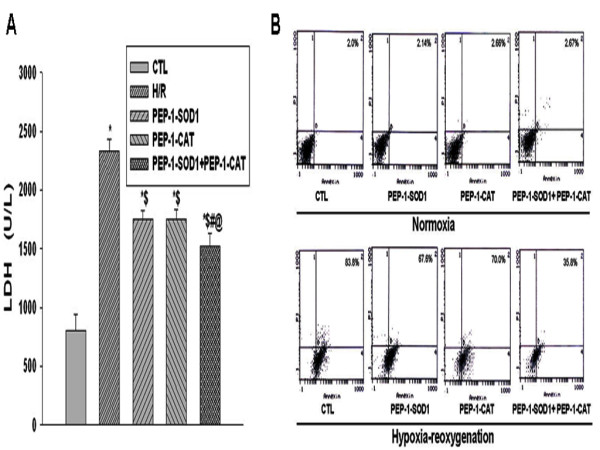
**Effect of PEP-1-SOD1 and PEP-1-CAT on LDH level and apoptosis rate**. (A) Effect of PEP-1-SOD1 and PEP-1-CAT on LDH level. *P < 0.01 vs. control (CTL) group; ^$^*P *< 0.01 vs H/R group; ^#^*P *< 0.01 vs PEP-1-SOD1 group; ^@^*P *< 0.01 vs PEP-1-CAT group (n = 5). (B) Effect of PEP-1-SOD1 and PEP-1-CAT on apoptosis of H9c2 cells under hypoxia-reoxygenation injury. H9c2 cells were pretreated with PEP-1-CAT for 6 h and/or PEP-1-SOD1 for 1 h. The cells were then placed in a normoxia environment for 27 h or in hypoxia chamber for 21 h followed by 6 h of reoxygenation. Apoptosis was measured by staining the cells with Annexin V and PI followed by Flow cytometry. The apoptosis rates are shown.

LDH level is an indicator of cellular injury. Compared to H/R group, LDH levels were decreased in PEP-1-SOD1 or PEP-1-CAT-treated groups. However, the reduction of LDH levels was greater in the groups with both PEP-1-SOD1 and PEP-1-CAT, as compared to individual protein-treated groups (Figure 3A).

In the normoxia environment, H9c2 cells apoptosis was not significantly different among pretreatment with PEP-1-SOD1 and/or PEP-1-CAT and control group. However, apoptosis rate of the control cells increased to 83.8% after treated with hypoxia-reoxygenation. The apoptosis was significantly reduced in cells treated with PEP-1-SOD1 or PEP-1-CAT. Combined use of PEP-1-SOD1 and PEP-1-CAT further inhibited the apoptosis (Figure 3B).

### PEP-1-SOD1 and PEP-1-CAT suppressed CK, CK-MB, cTnT and MDA levels in vivo

**Figure 4:**
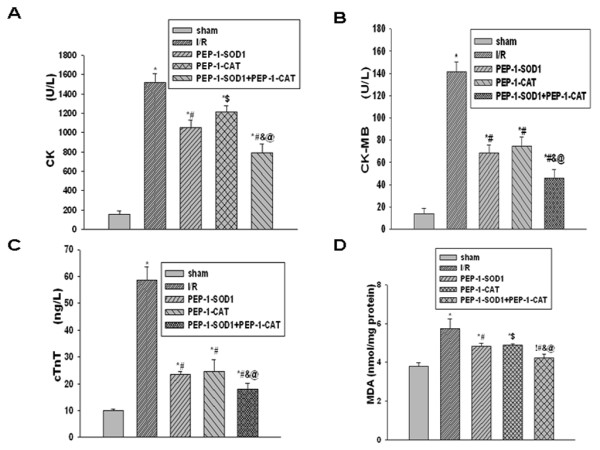
**Effects of PEP-1-SOD1 and PEP-1-CAT on CK, CK-MB, cTnT and MDA content after myocardial ischemia-reperfusion**. CK activity (A) and MDA levels (D) were measured as described in Materials and Methods. CK-MB activity (B) and cTnT (C) levels were quantified by ELISA. *P < 0.01 and ^!^*P *< 0.05 vs sham group; ^#^*P *< 0.01 and ^$^*P *< 0.05 vs I/R group; ^&^*P *< 0.05 vs PEP-1-SOD1 group; ^@^*P *< 0.01 vs PEP-1-CAT group. n = 6.

The animal survival rate after surgery in different groups was as follows: 100% in sham group, 57.7% in I/R group, 66.1% in PEP-1-SOD1+PEP-1-CAT group, 61.5% in PEP-1-CAT group, and 59.3% in PEP-1-SOD1 group. The activities of serum CK, CK-MB and cTnT were used to monitor the myocardial damage. MDA levels reflect cardiomyocyte oxidative damage. Compared to the sham group, CK, CK-MB activity, cTnT and MDA levels were markedly increased due to ischemia-reperfusion injury, but decreased after PEP-1-SOD1 or PEP-1-CAT treatment. Importantly, combined usage of PEP-1-SOD1 and PEP-1-CAT further suppressed CK, CK-MB activity, cTnT and MDA levels (Figure 4).

### PEP-1-SOD1 and PEP-1-CAT altered the expression of apoptosis proteins in vivo

**Figure 5:**
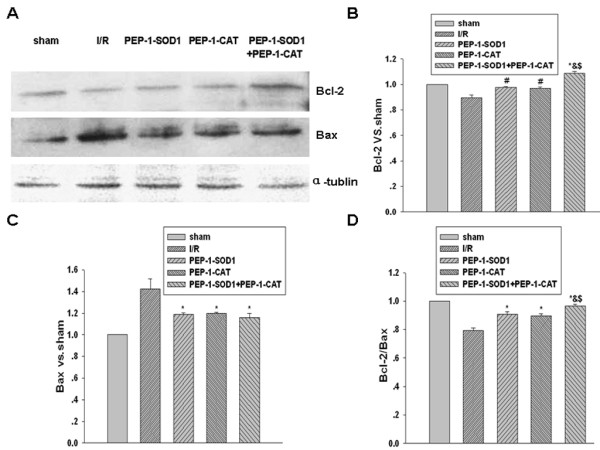
**Effect of PEP-1-SOD1 and PEP-1-CAT on Bcl-2 and Bax expression**. Bcl-2 and Bax expression was detected by Western blot as described in Materials and Methods (A), and quantified by normalization to tubulin (B and C). Bcl-2/Bax ratio (D) was calculated by dividing the normalized expression of Bcl-2 by Bax. *P < 0.01 and ^#^*P *< 0.05 vs I/R group; ^&^*P *< 0.05 vs PEP-1-SOD1 group; ^$^*P *< 0.01 vs PEP-1-CAT group. n = 6.

Bcl-2, an anti-apoptotic protein, promotes cell growth, while Bax, a pro-apoptotic protein member of Bcl-2 family, accelerates apoptosis. Western blot analysis showed that Bcl-2 expression was markedly increased, while Bax expression was markedly decreased in PEP-1-SOD1 or PEP-1-CAT-treated hearts (*P *< 0.05), as compared to I/R group (*P *< 0.05). Bcl-2 expression was further increased by the treatment with both PEP-1-SOD1 and PEP-1-CAT although Bax expression seems no significant changes. However, Bcl-2/Bax ratio in PEP-1-SOD1 and PEP-1-CAT-treated groups was significantly larger than the treatment with individual proteins (Figure 5). These data suggest that combination of PEP-1-SOD1 and PEP-1-CAT further inhibited ischemia-reperfusion-induced apoptosis.

### PEP-1-SOD1 and PEP-1-CAT decreased infarct size and improved left ventricular (LV) function

**Figure 6:**
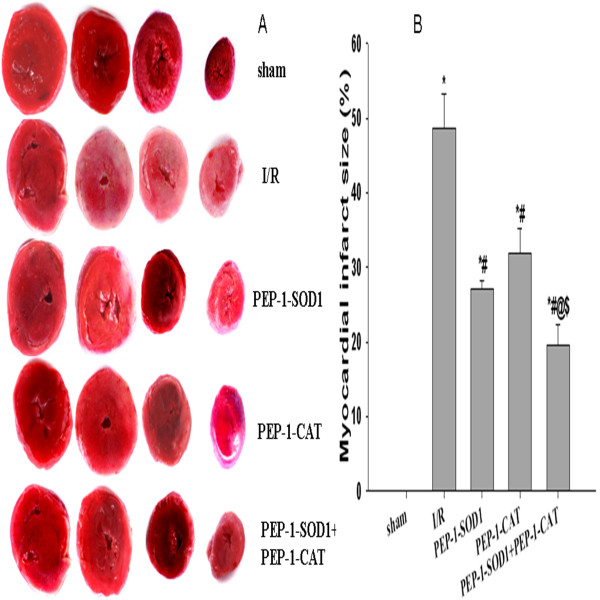
**PEP-1-SOD1 and PEP-1-CAT fusion proteins reduced myocardial infarction size**. (A) TTC-stained myocardium 2 h after reperfusion. (B) Infarction size in each group. The infarcted volume was expressed as a percentage of left ventricular volume for each heart. *P < 0.01 vs sham group; ^# ^*P *< 0.01 vs I/R group; ^@^*P *< 0.05 vs PEP-1-SOD1 group; ^$^*P *< 0.01 vs PEP-1-CAT group. n = 6.

To investigate whether the transduced PEP-1-SOD1 and PEP-1-CAT fusion proteins are biologically active *in vivo*, we measured the effects of PEP-1-SOD1 and PEP-1-CAT on myocardial infarct size with 1% TTC staining of the rat hearts with myocardial ischemia-reperfusion injury. Compared to I/R group (48.56 ± 4.63%), infarct size were reduced in rats pretreated with PEP-1-SOD1 (27.14 ± 4.10%) or PEP-1-CAT (30.12 ± 4.78%). The combined usage of both PEP-1-SOD1 and PEP-1-CAT had a much greater effect on the reduction of the necrotic area (20.38 ± 3.86%) (Figure 6). These results indicate that combination of PEP-1-SOD1 and PEP-1-CAT can more effectively decrease infarct size.

**Figure 7:**
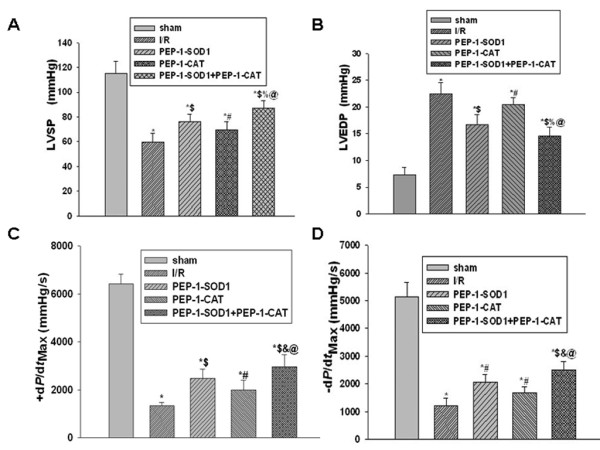
**Effect of PEP-1-SOD1 and PEP-1-CAT on hemodynamics**. 2 h after reperfusion, LV function was measured as described in Materials and Methods. LVSP-left ventricle systolic pressure (A); LVEDP-left ventricle end-diastolic pressure (B); ± d*p*/d*t*_max_-rate of the rise or fall of left ventricular pressure (C and D). *P < 0.01 vs sham group; ^#^*P *< 0.05 and ^$^*P *< 0.01 vs I/R group; ^&^*P *< 0.05 and ^%^*P *< 0.01 vs PEP-1-SOD1 group; ^@^*P *< 0.01 vs PEP-1-CAT group. N = 6.

*In vivo *hemodynamic measurements showed that the LV function was significantly improved in hearts treated with PEP-1-SOD1 or PEP-1-CAT compared to I/R hearts. Treatment with both PEP-1-SOD1 and PEP-1-CAT resulted in a larger increase of LVSP and ± d*p*/d*t*_max_, and a lower LVEDP compared to PEP-1-SOD1 or PEP-1-CAT individually-treated hearts (Figure 7).

## Discussion

Ischemic heart disease, a major cause of mortality in developed countries, is characterized by interrupted blood supply to the myocardium that leads to tissue necrosis. The treatment of this condition allows the rapid return of blood flow to the ischemic zones of the myocardium. However, reperfusion may cause further complications such as decreased cardiac contractile function and arrhythmias. Therefore, the developments of cardioprotective agents, which may delay the onset of necrosis during ischemia-reperfusion, lessen the necrotic tissue mass, improve myocardial function and decrease the incidence of arrhythmias is of great clinical relevance. The exact cellular mechanisms of ischemia-reperfusion injury are still a question of debate, however, among several other mediators, superoxide (O_2_^-^), nitric oxide (NO), and peroxynitrite (ONOO^-^) play a major role in ischemia-reperfusion injury [17,18]. Furthermore, there is good evidence that OFR, such as superoxide anions, hydroxyl radicals and hydrogen peroxide, mediate pathphysiology of human diseases [19-21]. OFR also prompts vascular smooth muscle cell migration and proliferation causing intimal hyperplasia and remodeling, eventually leading to artery restenosis after arterial balloon angioplasty [22]. A few studies suggest that there is continuous OFR-mediated oxidative stress injury after acute myocardial infarction treated by percutaneous coronary intervention (PCI) [23]. Another study shows that overexpression of Cu/Zn-SOD and/or catalase in ApoE-deficient mice suppresses benzo (a) pyrene-accelerated atherosclerosis [24]. Gene therapy is considered to be a promising approach, but some key problems of gene therapy have not been fundamentally resolved, including the efficiency of gene transfer, control of gene expression, effectiveness, security. Therefore, it is important to find new ways to modify antioxidant enzyme for the efficient introduction into cells.

The antioxidant enzymes (SOD1 and CAT) have the potential to prevent OFR-mediated tissue damage, but they cannot freely pass the cell membrane, which limits their applications. In this study, the human SOD1 and CAT gene were fused with a PEP-1 peptide to produce PEP-1-SOD1 and PEP-1-CAT fusion proteins. These fusion proteins can be transduced into cells and maintain their enzymatic activities. Our *in vitro *studies demonstrate that PEP-1-SOD1 and PEP-1-CAT together generate greater inhibitory effects than individual proteins on LDH release and apoptosis rates in cardiomyocyte H9c2 cells. The levels of LDH in the supernatants and the apoptosis of cells were indicators of hypoxia-reoxygenation injury [25,26].

Our previous studies have shown that application of PEP-1-SOD1 or PEP-1-CAT can tranduced into myocardium and protected against myocardial ischemia-reperfusion injury in rats [12,14]. To examine the combined effect of PEP-1-SOD1 and PEP-1-CAT, we applied both of PEP-1-SOD1 and PEP-1-CAT to rats with myocardial ischemia-reperfusion injury. CK CK-MB and cTnT are widely present in the cytoplasm of myocardial cells, and elevation of serum CK, CK-MB and cTnT are reliable indicators of myocardium injury [12,27]. MDA can be detected at a very early time of an injury, and is a reliable marker of myocardium oxidative damage. PEP-1-SOD1 or PEP-1-CAT reduced the increase of serum CK, CK-MB, cTnT and myocardial MDA levels caused by myocardial ischemia-reperfusion injury. However, combination of PEP-1-SOD1 and PEP-1-CAT resulted in a greater reduction of CK, CK-MB, cTnT and MDA, indicating that PEP-1-SOD1 and PEP-1-CAT cooperatively protected heart against ischemia-reperfusion injury by removing OFR.

Ischemia-reperfusion injury induces myocardial apoptosis [28,29]. OFR produced during the reperfusion turns on mitochondrial apoptosis pathway, which is considered to be the major mechanism of cardiomyocyte apoptosis [29,30]. Ligation of the left anterior descending coronary artery in dogs for 1 hour then 6~72 hours reperfusion have resulted in a reduction Bcl-2 and increase of Bax expression with the corresponding myocardial apoptosis and myocardial infarction [31]. These data suggest that the levels of regional myocardial expression of Bcl-2 and Bax after myocardial ischemia-reperfusion reflect the severity of cardiomyocyte apoptosis. Increased Bcl-2/Bax ratio may reduce cardiomyocyte apoptosis. PEP-1-SOD1 or PEP-1-CAT increased of Bcl-2 and Bcl-2/Bax ratio while reduced Bax levels. Combination of PEP-1-SOD1 and PEP-1-CAT further increased Bcl-2 level and Bcl-2/Bax ratio, suggesting that PEP-1-SOD1 and PEP-1-CAT combination may better prevent heart from myocardial ischemia-reperfusion-induced injury.

In addition, myocardial infarction area is correlated with the exercise tolerance capability. The smaller the infarction area, the better quality of life. Infarction areas in hearts treated with both PEP-1-SOD1 and PEP-1-CAT were significantly decreased compared to PEP-1-SOD1 or PEP-1-CAT-treated alone. Functionally, LVSP and ± *d*p/*d*t_max _were better improved, and LVDEP was further reduced with both PEP-1-SOD1 and PEP-1-CAT. Importantly, the LV function was also better improved with combined treatment of PEP-1-SOD1 and PEP-1-CAT.

The greater protective effects of combined use of PEP-1-SOD1 and PEP-1-CAT against myocardial ischemia-reperfusion injury are due to the combined function of SOD1 and CAT. PEP-1-SOD1 or PEP-1-CAT alone can only remove part of OFR. Combination of PEP-1-SOD1 and PEP-1-CAT not only more effectively and completely remove O_2_^- ^or H_2_O_2_, thus produce more oxygen to prevent oxygen deficiency, but also reduce myocardial apoptosis by blocking apoptotic factors such as CK, CK-MB, cTnT, LDH, MDA, which minimize the myocardial infarction, leading to an improved LV function.

## Conclusion

Combination of PEP-1-SOD1 and PEP-1-CAT fusion proteins can more efficiently protect against ischemia-reperfusion-induced myocardial injury than PEP-1-SOD1 or PEP-1-CAT alone, which provides a basis for using PEP-1-SOD1 and PEP-1-CAT together to prevent myocardial ischemia-reperfusion injury. This study provides valuable information for myocardial protection in acute myocardial infarction after percutaneous coronary intervention, cardiopulmonary bypass or heart transplantation.

## Competing interests

The authors declare that they have no competing interests.

## Authors' contributions

GQH designed and performed the experiments, collected the data and analyzed the results. JNW and JMT participated in the experimental design and interpretation of the results. LZ performed some of the *in vitro *experiments. FZ carried out Western blot. JYY participated in animal experiments. LYG made fusion protein and evaluated the apoptosis by Flow Cytometry. YL and SYC analyzed the results and help writing the manuscript. All the authors have read and approved the final manuscript.
